# Intimate partner violence is associated with HIV infection in women in Kenya: A cross-sectional analysis

**DOI:** 10.1186/1471-2458-13-512

**Published:** 2013-05-28

**Authors:** Chyun-Fung Shi, Fiona G Kouyoumdjian, Jonathan Dushoff

**Affiliations:** 1 Department of Biology, McMaster University, Hamilton, ON L8S 4K1, Canada; 2 Dalla Lana School of Public Health, University of Toronto, Toronto, ON M5S 2J7, Canada

**Keywords:** Intimate partner violence, Generalized linear mixed models, DHS, HIV, Kenya

## Abstract

**Background:**

The relationship between intimate partner violence (IPV) and women’s risk of HIV infection has attracted much recent attention, with varying results in terms of whether there is an association and what the magnitude of association is. Understanding this relationship is important for HIV surveillance and intervention programs.

**Methods:**

We analyzed data from the 2008-2009 Demographic and Health Survey (DHS) in Kenya, on 1,904 women aged 15-49. A generalized linear mixed model was adapted to explore the relationship between IPV and HIV prevalence, controlling for sociodemographic variables, and treating DHS survey clusters, province and ethnicity as random effects. We used principal components analysis (PCA) to calculate a single IPV score for each woman. The effect of HIV risk behaviours on the association between IPV and HIV was also assessed.

**Results:**

Controlling for relevant sociodemographic factors, we found that HIV risk was significantly associated with IPV (P <0.01). After adjustment for risk factors as well as sociodemographic variables, the positive association between IPV and HIV remained significant (P=0.035). The estimated effect size of this model corresponds to an odds ratio of 1.55 for HIV infection comparing a woman who experienced no IPV and a woman at the 95th percentile for our IPV index.

**Conclusion:**

This study provides further evidence that IPV and HIV are associated. In addition, we found that this association remains even when we controlled for several HIV risk factors. This implies that IPV can be used as a marker of potential HIV risk, and may be causally associated with HIV risk. Further, these results suggest that IPV monitoring and prevention may have a useful role in HIV prevention in Kenya. Further research, ideally based on longitudinal observations, is needed to disentangle these relationships.

## Background

Domestic violence against women is a worldwide phenomenon [1,2]. In an international study, self-reported lifetime experience of domestic violence against women ranged from 15% to 71% across 10 countries [3]. Domestic violence, and intimate partner violence (IPV) in particular, is a risk factor for many adverse physical and psychological health outcomes [2-11]. IPV is also related to behaviors which increase the risk of HIV acquisition, such as alcohol consumption [4,12-14]; inconsistent condom use [15-18]; concurrent partnerships [13,19-21]; and a larger number of sex partners [13,22]. Furthermore, women with a history of IPV may be less likely to access health care opportunities, including HIV testing [20]. The relationship between IPV and HIV risk in women is potentially important, and not yet well understood.

Several studies have found associations between various forms of IPV and HIV status. A cross-sectional survey of women at antenatal clinics in South Africa found that women’s HIV status was associated with joint exposure to both physical and sexual IPV [23]. A prospective study of gender power inequity in South Africa indicated that physical and sexual IPV at baseline were independently associated with higher levels of subsequent HIV risk [24]. In Rwanda, psychological IPV (but not physical or sexual IPV) and the overall IPV experience of married women were found to be significantly correlated with HIV risk [25]. In India, married women had significantly higher HIV prevalence when exposed to both physical and sexual IPV, but not to physical IPV alone [26]; and a recent analysis found that abused wives had a higher HIV risk due to both a higher HIV infection rate among abusive husbands and an increased risk of HIV transmission within abusive relationships [27].

There are also studies that have not found a significant association between women’s IPV experience and their HIV risk. These include studies in South Africa [28], Tanzania [29], Kenya [30,31] and a DHS (the Demographic and Health Survey)-based study of the Dominican Republic, Haiti, India, Kenya, Liberia, Malawi, Mali, Rwanda, Zambia and Zimbabwe [32]. A prospective study of serodiscordant couples in seven East and Southern African countries (Kenya, Rwanda, Tanzania, Uganda, Botswana, South Africa and Zambia) also found no significant association between IPV and HIV seroconversion among HIV discordant couples, though identified a significant association between IPV and HIV prevalence [33].

It is not surprising that various studies have found differing results, as they differ in geographic setting and how the population was sampled, as well as in the variables used and the statistical approaches taken. It is also important to note that lack of statistical significance in a given context does not in itself provide evidence that an association is absent or even weak.

Kenya was chosen for this study for its relatively high HIV gender prevalence ratio (prevalence in women is around 1.9 times higher than that of men, higher than most population-based studies in Africa), and because there is evidence that IPV is considered culturally acceptable [34]; in particular, men and women report similar amounts of male-perpetrated domestic violence [30]; and intimate partners are the most common perpetrators of sexual violence [35]. In this study, we used the Kenya DHS’s 2008-2009 data to investigate the relationship between IPV and HIV, specifically to assess for an association, to define the magnitude of the association, and to explore the role of HIV risk behaviours in this relationship. We conducted two separate analyses of the association between HIV and IPV, first controlling only for socio-demographic variables, then adding HIV risk factors to the model. Although violence against men from their intimate partners may also be a concern, it is reported much less than violence against women [34], p.214 [35], so we focus on the latter here.

## Methods

### Sample selection

Funded by the US Agency for International Development (USAID), DHS has conducted national household surveys on health-related issues in more than 90 countries since 1984. The Kenya 2008-2009 DHS surveyed women aged 15 to 49 in selected households. In every second selected household, women were offered voluntary HIV testing [34], p. 8. Of the 4,418 women eligible for HIV testing, 8.2% refused to provide blood and 3.5% were not interviewed or were absent from the blood collection [34], p.212. One woman per household was selected to participate in a domestic violence module. Questions on IPV were administered to women completing the domestic violence module who had ever had an intimate partner, with questions asked about either the current or most recent partner [34], p. 254. Our sample for this study is women who are: currently married or living with someone, completed the IPV questions, and obtained HIV testing.

Women participating in the DHS survey gave their informed consent before data collection and before giving blood for HIV testing [34]. For the domestic violence survey, privacy was ensured before the interview was conducted [34]. The blood collection and analysis was based on a protocol developed by the DHS and revised and approved by the Scientific and Ethical Review Committee of the Kenya Medical Research Institute, and the National AIDS Control Council [34]. Permission for using the data for this study was authorized by ICF International, which coordinates the DHS.

### Measures

There were 12 IPV questions in the DHS IPV survey questionnaire, grouped into four IPV types: psychological, severe physical, less severe physical and sexual. The participants were asked if they had ever experienced any of these, and if so, about frequency in the last 12 months. Dried blood spots were collected during the survey and tested for HIV using ELISA (see [34], p. 10).

Basic sociodemographic factors analyzed in this study were those commonly considered in the aformentioned studies, and included age, education, working status (whether respondents are currently working), religion, geographic residence (urban/rural), wealth and age gap between the respondents and their male partners [13,20,24,25,27-29,33,36-40]. On the basis of theoretical considerations and previous research [23-28,41-46] (though limited by the data collected), we also selected certain HIV risk factors *a priori* to assess: partner’s alcohol consumption, condom use at last sex, partner’s number of other wives, respondent’s of sex partners with the last 12 months and in lifetime. In addition, location (cluster ID and province) and ethnicity were treated as random effects to properly control for correlations between people from the same geographic area and ethnicity. Women who reported no sexual activity within the preceding 12 months were not asked about condom use; we coded these women as “not asked” rather than excluding them from the study.

### Statistical analysis

We used generalized linear mixed models (GLMMs) to examine the association between IPV and HIV infection. The GLMM framework allows us to model a binary response variable (HIV test result), and to take random effects (location and ethnicity) into account. To simplify interpretation and to account for relationships between predictors, we assessed all the selected sociodemographic variables with the IPV predictor together in a single GLMM. Given theoretical and empirical uncertainty about whether HIV risk factors may mediate and/or confound the association between IPV and HIV, we included HIV risk factors in a model with sociodemographic variables to see whether and how they affected the magnitude of association between IPV and HIV, without presupposing which of these roles they might have.

To construct a simple model, we combined the 12 IPV questions (see Table 1) by converting responses into scores (see Table 2), and used the first principal component from a scaled, uncentered principal components analysis (PCA) as an index to describe the overall IPV experience of each woman, using a single variable.

**Table 1:** Responses to IPV questions

**IPV question**	**Proportion**
**Any less severe violence**	**34.5*%***
Spouse ever kicked or dragged	12.1%
Spouse ever punched with fist or something harmful	9.4%
Spouse ever pushed, shook or threw something	16.9%
Spouse ever slapped	29.9%
Spouse ever twisted her arm or pull her hair	7.7%
**Any psychological violence**	**27.9*%***
Spouse ever humiliated her	15.2%
Spouse ever insult or make feel bad	20.4%
Spouse ever threatened her with harm	14.1%
**Any severe violence**	**4.4*%***
Spouse ever threatened or attack with knife/gun	2.8%
or other weapon	
Spouse ever tried to strangle or burn	2.7%
**Any sexual violence**	**13.7*%***
Spouse ever physically forced sex when not wanted	13.3%
Spouse ever forced other sexual acts when not wanted	3.9%

Proportions are based on participants’ self-report on whether they experienced any IPV in their lifetime.

**Table 2:** Sociodemographic breakdown of HIV prevalence and IPV scores

	**N**	**HIV+**	**IPV score**
**Age 5-year groups**			
15-19	91	0.1648	2.25
20-24	422	0.0616	2.93
25-29	427	0.0890	2.76
30-34	360	0.0722	3.48
35-39	257	0.0623	3.30
40-44	181	0.0718	3.30
45-49	166	0.0542	3.28
**Highest educational level**			
No education	338	0.0414	3.080
Primary	1042	0.0883	3.501
Secondary	390	0.0692	2.727
Higher	134	0.0746	0.806
**Wealth index**			
Poorest	437	0.0412	3.67
Poorer	304	0.0954	3.60
Middle	313	0.0479	2.98
Richer	359	0.0724	2.95
Richest	491	0.1120	2.39
**Religion**			
Roman Catholic	371	0.0836	3.66
Protestant/ other Christian	1127	0.0870	3.13
Muslim	336	0.0268	2.13
None/Other	70	0.0714	3.70
**Type of place of residence**			
Urban	514	0.1128	2.68
Rural	1390	0.0612	3.22
**Respondent currently working**			
No	798	0.0551	2.66
Yes	1106	0.0895	3.38
**Current marital status**			
Married	1763	0.0715	3.05
Living together	141	0.1206	3.45

Raw IPV scores were coded as follows: women who reported never experiencing IPV are coded as 0, and others were coded based on reported IPV in the last 12 months: none=1, sometimes=2 and often=3, frequency missing (includes those with no partner in the last 12 months) = 1.5. The PCA-based IPV index was scaled from 0 to 36, so it is roughly comparable to the raw score.

We made an *a priori* decision to model wealth effects using a three-knot spline, and age effects using a four-knot spline.

In a follow-up model, we constructed a separate PCA index for each of the four DHS categories of IPV, using the same methodology as we used for the overall index.

Variable-level p values were calculated by sequentially dropping each variable and comparing the restricted models to the original model.

### Scripts

We are not able to make our data available, but researchers can request them from DHS. All of the R scripts use to analyze the data and produce the figures are available for download at http://lalashan.mcmaster.ca/theobio/Kenya_IPV_risk/.

## Results and discussion

### Data set

The analyses included 1904 women, after dropping those who were not currently in a relationship, and those with missing data. Their overall HIV prevalence was 7.5%, compared to 8.3% for the whole national DHS survey, and 6.3% for the 350 otherwise-eligible women who were not selected for the domestic-violence module.

Table 2 presents the four types of IPV categorized in the DHS surveys, their sub-categories, and the proportion of women in our sample reporting each type of IPV. Less severe physical IPV is the most common type of IPV experienced, followed by psychological IPV, sexual IPV and severe physical IPV.

Sociodemographic variables and their relationships with HIV prevalence and IPV (measured by the first component of a PCA decomposition, see Methods) are shown in Table 1.

### Models

When controlling for the sociodemographic variables only (the base model), there was a strongly significant positive association between IPV and HIV status Table 3. The estimated slope of the response of logistic predictor to our IPV index was 0.047; this means that, compared to a zero-IPV baseline, the odds ratio for HIV risk of a woman experiencing the mean amount of IPV was 1.13 and that of a woman at the 95th percentile for IPV was 1.79. Of the sociodemographic variables, only working status was a significant predictor of HIV in the multivariate model: women reported currently working were more likely to be HIV positive (OR estimate 1.66, P=0.016). Effect sizes for all variables in the model are shown in Additional file 1: Table S1 (fixed effects) and Additional file 2: Table S2 (random effects). When controlling for risk factors as well as sociodemographic variables in the full model, the significant positive association between IPV and HIV risk remained (see Table 3). The estimated slope of the response of logistic predictor to our IPV index was 0.036. The results mean that, compared to a zero-IPV baseline, the odds ratio of HIV risk was 1.10 for a woman experiencing the mean amount of IPV and that of a woman at the 95th percentile for IPV was 1.55. The effect of working status remained the only significant sociodemographic predictor (OR estimate 1.62, P=0.023). Of the HIV risk factors, only lifetime number of partners was significant in the multivariable model. The estimated OR for an additional lifetime partner was 1.21 (P=0.014).

**Table 3:** P values for the variables in the base model and the full model

	**df**	**P(*χ***^**2**^**)**	
		**Base**	**Full**
IPV index	1	0.003*	0.035*
Age	4	0.332	0.140
Religion	3	0.535	0.694
Edu	3	0.164	0.284
Urban/rural	1	0.143	0.148
Wealth score	3	0.210	0.281
Employment	1	0.016*	0.023*
Age gap	3	0.054	0.138
Number of partners (year)	1	—	0.182
Number of partners (lifetime)	1	—	0.014*
Condom	2	—	0.068
Other wives	1	—	0.099
Male alcohol	1	—	0.064

**Figure 1:**
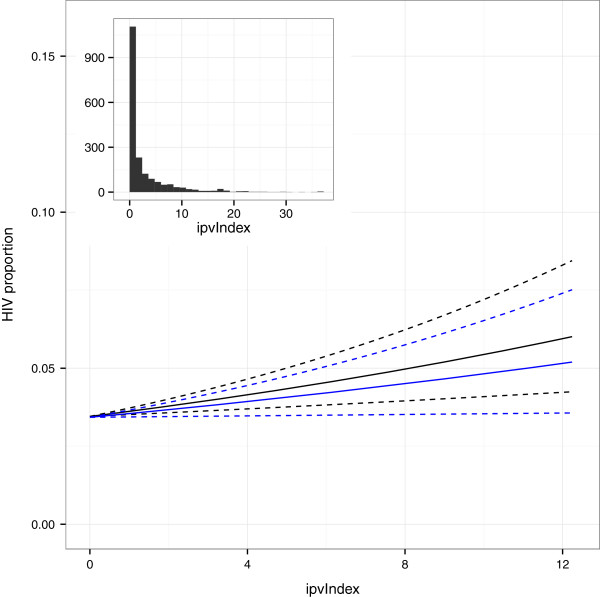
**Estimated effect on HIV infection of various levels of IPV from the base model (black) and the full model (blue).** Dashed lines show 95% confidence intervals for size of the effect (using no reported IPV as a baseline for comparison). For clarity of display, the x-axis is truncated at a value corresponding to the 95th percentile of the IPV index (see inset).

Figure 1 shows the estimated effect on HIV infection of various levels of IPV, compared to a relationship with no IPV. Other factors held equal, women who report IPV were more likely to test positive for HIV than those who did not, when controlling for sociodemographic variables (black lines), or sociodemographic variables and risk factors (blue lines). The estimated effect of IPV on HIV risk is similar, but not as strong, when risk factors are added to the model.

When we replaced our IPV index with separate indices for each of the four DHS categories of IPV, the response to socio-demographic variables and risk factors remained the same, but none of the individual categories indicated a statistically significant response.

An earlier version of our model used a quadratic, rather than linear, response of HIV risk to IPV. We discarded this model because it presented similar significance levels to the current version, but was more difficult to interpret.

### Discussion

Based on a nationally representative sample, we have identified a positive association between a PCA-based index of overall IPV and HIV infection, after controlling for basic sociodemographic variables. This association persists when HIV risk factors are included in the model. Working status and reporting a larger number of lifetime sexual partners were also positively associated with women’s HIV risk. Our study combines four types of IPV: psychological, less severe physical, severe physical, and sexual IPV. This comprehensive approach may allow a more accurate estimation of the association between IPV and HIV. A follow-up model that tested the four categories of IPV separately did not find a significant effect of any category, which may be due to a lack of sufficient power.

A bivariate analysis of data on people attending an STI clinic in Nairobi, Kenya [37] found a similar association, while other Kenyan studies did not find any association [30-32]. Of particular interest, a prospective study found no significant association between IPV and HIV seroconversion in discordant couples, though they did find a significant association between IPV and prevalent HIV infection [33]. This study examined data from seven nations in East and Southern Africa and did not report data stratified by country, which may be problematic if there is geographic variation in the magnitude or very existence of the association between IPV and HIV. These differing results may in part be due to the fact that different studies analyzed data from different population subgroups (e.g. pregnant women [31] and couples [30,33] in clinical sites), examined fewer or different types of IPV [30,32,33,37] than our study, and used univariate analysis [37] or different sets of basic and risk-factor variables.

Our study has several limitations. Our reliance on cross-sectional data precludes direct investigation of causal relationships. Some relevant potential confounders were not available, such as partner’s sexual risk behaviours and partner’s HIV status. Another potential source of bias is the self-selection of individuals for both of the violence module and the DHS HIV screen. Participants may also underreport their experience of IPV due to potential stigma. There may also be error in the measurement of sociodemographic and HIV risk behaviours, as well as in IPV.

Although there are programs to test for HIV during counseling for gender-based violence in Kenya [47] and the government passed a Sexual Offenses Act to penalize sexual assault [48], enforcement of the Act is a challenge because of fear among victims and perceived stigma from the community, and domestic violence is not yet formally recognized by law [49].

Although our cross-sectional study does not provide direct evidence that IPV contributes to HIV risk, our findings are consistent with several hypothesized mechanisms for a causal association between IPV and HIV, including that women who experience IPV may be unable to negotiate safer sexual behaviours such as condom use [15-18], may have riskier sexual behaviours [4,13,19-22], or may have relatively compromised immune systems due to the stress of IPV [7]. Additionally, it is possible that sexual IPV may directly lead to HIV infection. Causality in the other direction – i.e. that women with HIV may be more likely to experience IPV [33,50] – is also possible. Regardless of causality, the finding of association is important: knowing that IPV is associated with HIV risk could be useful for secondary prevention in nations where both IPV and HIV prevalence is high.

## Conclusion

In most of sub-Saharan Africa, HIV prevalence is higher in women than in men, suggesting that HIV intervention programs can benefit from a gendered perspective. Our findings suggest that IPV screening can aid in HIV intervention programs. Women who experience IPV should be considered to be at high risk for HIV (if they haven’t been tested), and should be considered for follow-up HIV testing. IPV screening and prevention programs would have dual benefits, since they could possibly reduce HIV transmission, as well as providing resources to women experiencing violence. IPV screening and intervention must be implemented in a culturally appropriate way, however, especially in a society where violence is culturally accepted [30]. Further research, including large-scale longitudinal studies with data on couples, is needed to elucidate the causal relationships between IPV and HIV.

## Competing interests

The authors declare no competing interests.

## Authors’ contributions

CS conceived the study. All three authors designed the statistical analyses, conducted by JD. All three authors interpreted the results. CS wrote the first draft of the manuscript. All authors revised the manuscript for important intellectual content, and approved final submission.

## Pre-publication history

The pre-publication history for this paper can be accessed here:

http://www.biomedcentral.com/1471-2458/13/512/prepub

## Supplementary Material

Additional file 1 **Table S1.** Effect sizes and standard errors for the fixed effects in the base model and the full model. Click here for file

Additional file 2 **Table S2.** Effect standard deviations for the random effects in the base model and the full model. Click here for file
