# Supplementary material for: Intimate partner violence is associated with HIV infection in women in Kenya: A cross-sectional analysis
Source: BMC Public Health. 2013 May 28;13:512. doi: 10.1186/1471-2458-13-512 (PMC3702473; doi:10.1186/1471-2458-13-512)
Supplement: Additional file 1 — Table S1. Effect sizes and standard errors for the fixed effects in the base model and the full model. [file 1471-2458-13-512-S1.pdf]

Table S1 Effect sizes and standard errors for the fixed effects in the base model and the full model

	Base		Full	
	**Estimate**	**SE**	**Estimate**	**SE**
IPV index	0.04743	0.01509	0.03551	0.01621
age (splined)				
component 1	-0.54443	0.58223	-0.63539	0.59051
component 2	-1.02540	0.57724	-1.27208	0.59491
component 3	-2.63396	1.40718	-3.01919	1.43845
component 4	-0.33521	0.49996	-0.57664	0.52675
religion (baseline, Catholic)				
Christian	-0.06632	0.23405	0.03557	0.24213
Muslim	-0.47225	0.48921	-0.29248	0.49063
None/Other	0.46731	0.58514	0.49063	0.59453
education (baseline, None)				
Primary	-0.45471	0.41505	-0.45464	0.41493
Secondary	-0.91526	0.47662	-0.85293	0.48101
Higher	-0.88158	0.57200	-0.76530	0.58494
rural (vs. urban)	-0.43553	0.29732	-0.44199	0.30571
Wealth (splined)				
component 1	1.11960	0.77732	1.07358	0.78005
component 2	1.31175	2.87058	0.89175	2.85478
component 3	0.86579	0.93091	0.53757	0.94813
working (yes)	0.50700	0.21297	0.48532	0.21630
age gap(splined)				
component 1	-0.34023	0.44475	-0.33733	0.45730
component 2	-2.99492	1.20215	-2.67019	1.25094
component 3	-0.22889	0.47735	-0.23690	0.49898
number of partners (year)			-1.23828	1.08831
number of partners (lifetime)			0.18925	0.07482
Condom usage (baseline, No)				
Yes			0.91431	0.37804
Not asked			-0.45889	1.20085
other wives			0.21083	0.12198
male alcohol			0.37932	0.20293
