# Supplementary material for: Intimate partner violence is associated with HIV infection in women in Kenya: A cross-sectional analysis
Source: BMC Public Health. 2013 May 28;13:512. doi: 10.1186/1471-2458-13-512 (PMC3702473; doi:10.1186/1471-2458-13-512)
Supplement: Additional file 2 — Table S2. Effect standard deviations for the random effects in the base model and the full model. [file 1471-2458-13-512-S2.pdf]

Table S2 Effect standard deviations for the random effects in the base model and the full model

	**Base**	**Full**
clusterId	0.2270	0.2732
ethnicity	0.7539	0.6825
province	< 0.001	< 0.001
